# Genomic Analysis of Diverse Members of the Fungal Genus *Monosporascus* Reveals Novel Lineages, Unique Genome Content and a Potential Bacterial Associate

**DOI:** 10.1534/g3.120.401489

**Published:** 2020-06-24

**Authors:** Aaron J. Robinson, Donald O. Natvig, Patrick S. G. Chain

**Affiliations:** ∗ Bioscience Division, Los Alamos National Laboratory, Los Alamos, New Mexico 87545, and; ^†^ Department of Biology, University of New Mexico, Albuquerque, New Mexico 87131

**Keywords:** *Monosporascus*, fungal genomics, bacterial associates, *Ralstonia*

## Abstract

The genus *Monosporascus* represents an enigmatic group of fungi important in agriculture and widely distributed in natural arid ecosystems. Of the nine described species, two (*M. cannonballus* and *M. eutypoides*) are important pathogens on the roots of members of Cucurbitaceae in agricultural settings. The remaining seven species are capable of colonizing roots from a diverse host range without causing obvious disease symptoms. Recent molecular and culture studies have shown that members of the genus are nearly ubiquitous as root endophytes in arid environments of the Southwestern United States. Isolates have been obtained from apparently healthy roots of grasses, shrubs and herbaceous plants located in central New Mexico and other regions of the Southwest. Phylogenetic and genomic analyses reveal substantial diversity in these isolates. The New Mexico isolates include close relatives of *M. cannonballus* and *M. ibericus*, as well as isolates that represent previously unrecognized lineages. To explore evolutionary relationships within the genus and gain insights into potential ecological functions, we sequenced and assembled the genomes of three *M. cannonballus* isolates, one *M. ibericus* isolate, and six diverse New Mexico isolates. The assembled genomes were significantly larger than what is typical for the Sordariomycetes despite having predicted gene numbers similar to other members of the class. Differences in predicted genome content and organization were observed between endophytic and pathogenic lineages of *Monosporascus*. Several *Monosporascus* isolates appear to form associations with members of the bacterial genus *Ralstonia* (Burkholdariaceae).

Members of the fungal genus *Monosporascus* (Ascomycota, Sordariomycetes, Xylariales) are known for their association with plant roots in agricultural and natural arid environments. Most research focused on the genus has addressed aspects of pathogenicity in agricultural settings, with the result that little is known about the functional and evolutionary diversity of members of this genus in natural environments. To gain deeper insight into the diversity and evolutionary history of *Monosporascus*, and to evaluate whether lineages that possess agricultural pathogens differ from those that appear to be benign endophytes in natural environments, we generated the first complete genome sequences for members of the genus, targeting isolates from diverse environments and locations.

There are currently several recognized *Monosporascus* species, including the closely related *M. cannonballus and M. eutypoides*, which have been studied for their ability to cause disease in the roots of many agriculturally important members of the Cucurbitaceae (Ben Salem *et al.* 2013). A third species, *M. ibericus*, was described as an endophyte in the roots and stems of several plant species in Spain (Collado *et al.* 2002). Recent surveys conducted in northern Brazil also resulted in the description of five new *Monosporascus* species: *M. brasiliensis*, *M. caatinguensis*, *M. mossoroensis*, *M. nordestinus* and *M. semiaridus* (Negreiros *et al.* 2019). Diverse members of the genus are also common in root endophyte surveys conducted in the southwestern United States using both molecular and culturing methods (Porras-Alfaro *et al.* 2008; Herrera *et al.* 2010; Dean *et al.* 2015). *Monosporascus* isolates display a wide range of morphological phenotypes when cultured on potato dextrose agar (PDA) (Figure 1B). The near ubiquity of *Monosporascus* species on the roots of plants in arid Southwestern ecosystems raises questions about their evolutionary diversity and primary ecological roles. There is no evidence that isolates of *Monosporascus* from the roots of plants in natural ecosystems cause disease symptoms despite their broad host association, which raises the question: can differences in host interaction be explained by differences in genome evolution or content? This question is complex and possible explanations include the evolution of functionally different sub-lineages and environmentally-driven shifts in genome content. To examine this question, we employed methods that included comparisons of genome synteny and functional gene content. We were particularly interested in genes that may be associated with fungal-plant interactions and genomic regions that demonstrate a lack of synteny among *Monosporascus* assemblies.

**Figure 1:**
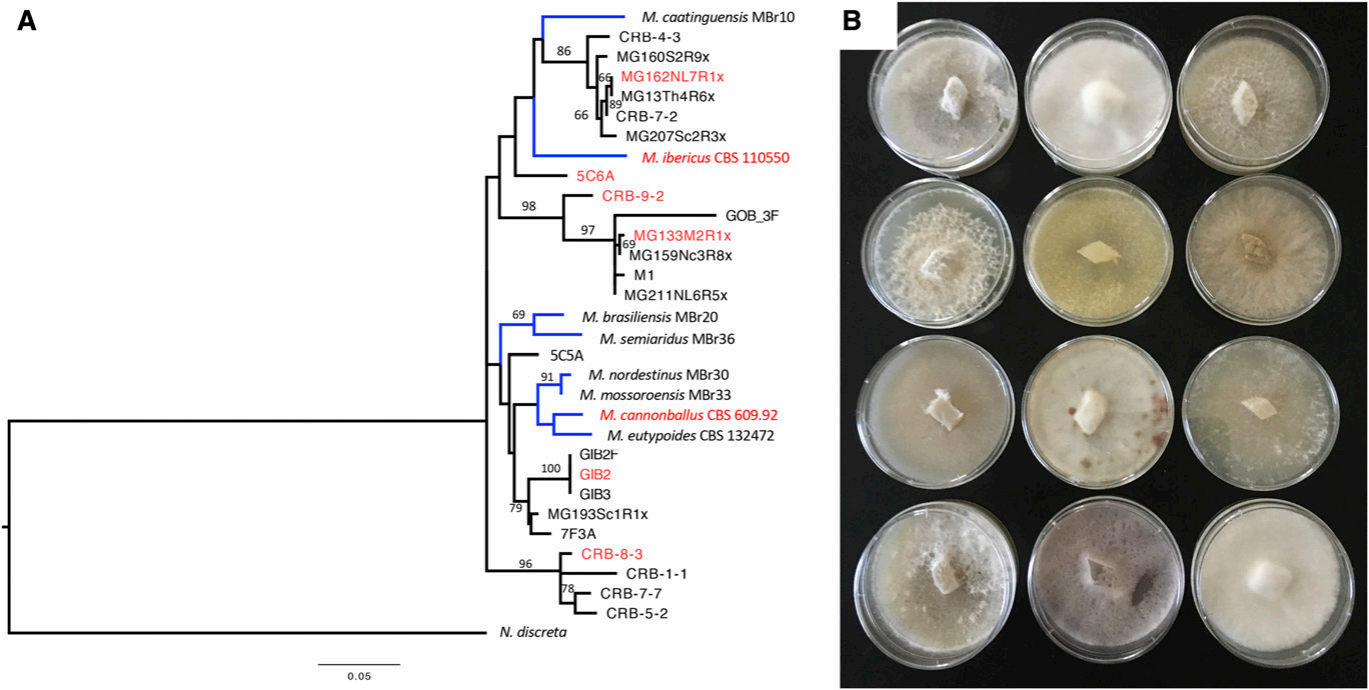
Diversity of *Monosporascus* isolates. (A) Maximum likelihood tree for strains of *Monosporascus* based on analysis of ribosomal RNA ITS sequences. Diversity among *Monosporascus* ITS sequences was used to select isolates for whole genome sequencing (sequenced isolates are highlighted in red). Previously described isolates of *Monosporascus* are indicated by branches shown in blue. Other isolates are from culture surveys of root endophytes at the SNWR in central New Mexico. Bootstrap values (percent of 1000 replicates) are shown for branches with greater than 65% support. (B) Diversity of *Monosporascus* isolates from the SNWR plated on potato dextrose agar (PDA).

As reported here, preliminary comparisons of internal transcribed spacer (ITS) DNA regions indicated that certain *Monosporascus* isolates from arid environments were closely related to previously described species, while other isolates appeared to represent novel lineages. In order to explore this diversity more deeply, we sequenced and assembled the genomes of ten *Monosporascus* isolates, including representatives of two described species from culture collections in addition to several recent isolates from New Mexico. This work represents the first genome sequences and assemblies for the genus. The genome sizes revealed by these assemblies are significantly larger than what is commonly found in the Xylariales and other Ascomycota. Despite their larger size, the *Monosporascus* genomes have gene numbers and genome organization similar to other members of the Xylariales. Phylogenetic analyses confirmed substantial diversity across the genus while aiding in understanding the relationship of *Monosporascus* species to other members of the Xylariales. The availability of complete genome assemblies also allowed for comparisons of genome content and organization between the agricultural and aridland *Monosporascus* isolates.

Although comparisons among genomes of isolates of *Monsoporascus* and other Xylariales genomes indicated substantial difference in genome organization across genera, comparisons of genomes among closely-related agricultural and aridland *Monosporascus* isolates revealed few differences in genome organization and content. Within the genus *Monosporascus*, endophytic strains possessed higher numbers of genes for predicted carbohydrate-active and pathogenesis enzymes than did pathogenic isolates. Across the Xylariales, however, there was no clear correlation between gene groups and primary ecological function. Members of the genus *Monosporascus* appear to have the capability to accommodate bacterial endosymbionts from the Burkholderiaceae.

## Materials And Methods

### Sample collection

*Monosporascus cannonballus* (CBS 586.93 & CBS 609.92) and *Monosporascus ibericus* (CBS 110550) strains were obtained from the CBS-KNAW fungal collection (http://www.westerdijkinstitute.nl/Collections/) (USDA permit P526P-16-01285). Six additional isolates were obtained from surface sterilized roots collected in previous endophyte surveys conducted at the Sevilleta National Wildlife Refuge (SNWR) in New Mexico (Porras-Alfaro *et al.* 2014; Dean *et al.* 2015,). *Monosporascus cannonballus* strain MC13-8B was provided courtesy of Michael Stanghellini, University of California, Riverside. Geographic and host information are provided in Table 1.

**Table 1:** Metadata and assembly statistics for *Monosporascus* isolates selected for whole genome sequencing

Species (strain)	Location	Host	NCBI Accession	Number of contigs	N50	Total length (Mb)
*Monosporascus cannonballus* (CBS 609.92)	Arizona	*Cucumis melo*	QJNS00000000	753	256,764	89.62
*Monosporascus cannonballus* (CBS 586.93)	Egypt	*Cucumis melo*	QJNT00000000	758	248,352	91.50
*Monosporascus cannonballus* (MC13-8B)	California	*Cucumis melo*	QJNW00000000	1,441	151,284	89.29
*Monosporascus ibericus* (CBS110550)	Spain	Undetermined	QJNU00000000	1,612	104,428	87.25
*Monosporascus* sp. (5C6A)	New Mexico	*Bouteloua eriopoda*	QJOB00000000	823	246,621	75.25
*Monosporascus* sp. (CRB-8-3)	New Mexico	*Larrea tridentata*	QJNZ00000000	2,382	93,740	102.96
*Monosporascus* sp.(CRB-9-2)	New Mexico	*Larrea tridentata*	QJOA00000000	2,348	88,305	76.10
*Monosporascus* sp. (MG133)	New Mexico	*Mentzelia perennis Wooton*	QJNX00000000	768	166,637	71.87
*Monosporascus* sp. (MG162)	New Mexico	*Nerisyrenia linearifolia*	QJNY00000000	1,320	134,088	89.46
*Monosporascus* sp. (GIB2)	New Mexico	*Bouteloua eriopoda*	QJNV00000000	935	138,727	70.59

### Molecular methods and sequencing

Genomic DNA was obtained from pure cultures following a CTAB extraction protocol (Robinson and Natvig 2019). The KAPA Hyper Prep Kit (Kapa Biosystems, Wilmington, Massachusetts) was used to prepare genomic libraries for each isolate. Genome sequencing was performed using the Illumina NextSeq 500 platform configured for 150 base-pair read lengths. A total yield of 52.99 Gbp was generated from this sequencing run. The CRB-9-2 isolate contained the smallest percentage of pass-filtered reads (4.02% or ∼1.07 Gbp), while the MC13-8B isolate contained the largest percentage of pass filtered reads (13.63% or ∼3.46 Gbp).

### Genome assembly

Quality control of the resulting Illumina reads was performed using Trimmomatic (Bolger *et al.* 2014), and three separate software packages designed for short read de-novo microbial genome assemblies were independently optimized to assemble these QC-filtered reads (Robinson and Natvig 2019). Quast (Gurevich *et al.* 2013) was used to generate assembly statistics, and additional assembly quality assessment was performed using the BUSCO software package and Ascomycota dataset (Simão *et al.* 2015). Assemblies produced in SPAdes (Bankevich *et al.* 2012) consistently showed the highest quality and were annotated using AUGUSTUS with *Neurospora crassa* selected for the species parameter, limited to few alternative transcripts and only complete gene predictions (Stanke and Morgenstern 2005; Hoff and Stanke 2013). These assemblies have been deposited at GenBank with the following accession numbers: QJNS00000000, QJNT00000000, QJNU00000000, QJNV00000000, QJNW00000000, QJNX00000000, QJNY00000000, QJNZ00000000, QJOA00000000, and QJOB00000000.

### Bioinformatics

The search function of the MMseqs2 (Steinegger and Söding 2017) software was used with default settings to compare predicted amino-acid sequences from our annotations of *Monosporascus* genomes. CD-HIT (Li and Godzik 2006) was used to examine our assemblies for gene duplications and to compare protein similarities among assemblies. Predicted protein sequences generated by AUGUSTUS were annotated for carbohydrate-active enzymes using the dbCAN2 meta server (http://bcb.unl.edu/dbCAN2/) Zhang* et al*. 2018 For the dbCAN2 annotation we utilized HMMER (E-Value < 1e-15, coverage > 0.35), DIAMOND (E-Value < 1e-102) and Hotpep (Frequency > 2.6, Hits > 6), and only candidates agreed upon by at least two of these methods were retained. A local version of the PHI-base (Urban *et al.* 2017) database was created using BLAST, and predicted protein sequences were queried against this database using blastp. Results were filtered to retain matches with e-value scores less than or equal to 1e-10 and only the top hit was used in downstream analysis. InterProScan Jones *et al*. (2014) was used to infer the function of protein sequences based on the presence of conserved domains from non-syntenic regions of *Monosporascus* and other members of the Xylariales using the top matches identified with default settings.

### Molecular alignments and phylogenetic analyses

Ribosomal RNA ITS sequences employed in preliminary phylogenetic analyses were obtained from our *Monosporascus* isolate collection and databases of the National Center for Biotechnology Information (NCBI). ITS sequences were aligned with Clustal Omega (Sievers *et al.* 2011) and RAxML (Stamatakis 2014) was used to construct maximum likelihood phylogenetic trees using the GTRCAT substitution model and 1000 bootstrap replicates. The GTRCAT model was automatically determined as the best-scoring nucleotide substitution model for this data by the RAxML software.

Subsequent to the acquisition of genome sequence data, evolutionary relationships among *Monosporascus* isolates were evaluated using whole-genome phylogenetic analyses performed with PhaME (Shakya *et al.* 2020). PhaME analyses were conducted with the reads used in the assembly of the ten *Monosporascus* genomes, employing the smallest (GIB2) assembly as the reference and 100 bootstrap replicates. To maximize the number of nucleotide positions available for comparisons, only the ten *Monosporascus* genomes were used.

### Data availability

Strains are available upon request. These assemblies have been deposited at GenBank with the following accession numbers: QJNS00000000, QJNT00000000, QJNU00000000, QJNV00000000, QJNW00000000, QJNX00000000, QJNY00000000, QJNZ00000000, QJOA00000000, and QJOB00000000. The NCBI BioProject accession for this work is PRJNA471727 and includes links to the whole genome sequencing data used for assemblies and phylogenetic analyses, which are available through the NCBI SRA database. The authors affirm that all other data necessary for confirming the conclusions of the article are present within the article, figures, and tables. Supplemental material available at figshare: https://doi.org/10.25387/g3.12494639.

## Results

### Genome sequencing and assembly

**Figure 2:**
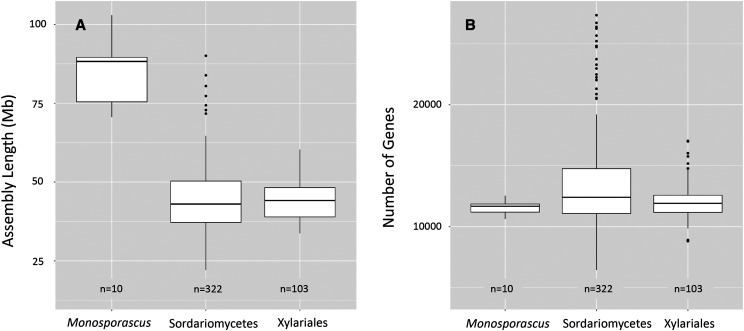
Comparison of genome assembly length and number of genes among the ten *Monosporascus* assemblies and other members of the Sordariomycetes and Xylariales represented at the JGI Mycocosm portal. (A) Genome assembly length (Mb) comparison based on the most current representative assemblies. (B) Number of predicted genes in the *Monosporascus* assemblies compared with other members of the Sordariomycetes and Xylariales.

**Figure 3:**
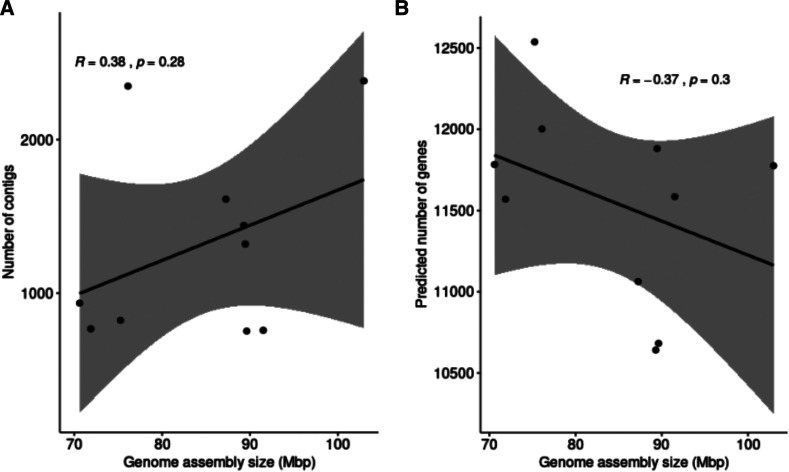
Correlation plots evaluating relationships among genome assembly and annotation statistics for the ten *Monosporascus* assemblies. Genome size was not significantly correlated with either contig number or gene number. (A) Genome assembly size *vs.* number of contigs. (B) Genome assembly size *vs.* predicted number of genes.

We sequenced, assembled and annotated the genomes of ten *Monosporascus* isolates that spanned the diversity of isolates based on ITS sequence analyses (Figure 1A). Data from the Joint Genome Institute (JGI) Mycocosm portal (https://mycocosm.jgi.doe.gov/mycocosm/home) indicate an average genome assembly length of 44.37 Mb for Sordariomycetes (322 genomes) and 44.31 Mb for Xylariales (103 genomes). The sizes of the *Monosporascus* genome assemblies were substantially larger than these averages, ranging from 70 to greater than 100 Mb (Figure 2A). Summary statistics for the final SPAdes assemblies demonstrated no relationship between assembly fragmentation (number of contigs) and total assembly size (Table 1 & Figure 3A).

**Figure 4:**
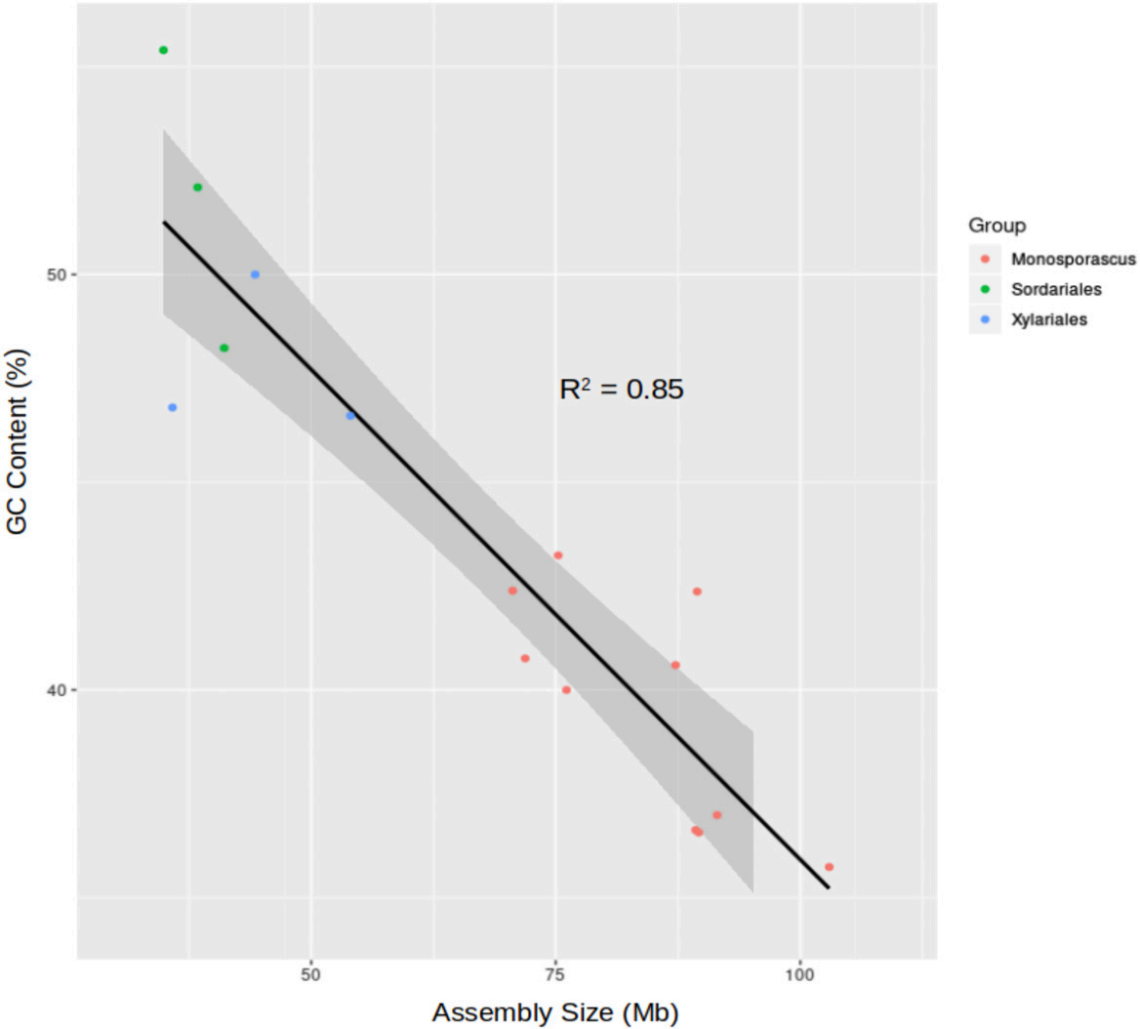
Relationship between GC content and genome size for several genomes from the Sordariales and Xylariales. Linear regression analysis demonstrating the strong negative relationship between GC content and assembly size (p-value = 2.701e-07). The ten *Monosporascus* assemblies are represented in red. Green circles indicate assemblies for the following members of the Sordariales: *Neurospora crassa* (41.1 Mb), *Sordaria macrospora* (38.39 Mb) and *Chaetomium globosum* (34.89 Mb). The blue circles indicate assemblies for the following members of the Xylariales: *Eutypa lata* (54.01 Mb), *Rosellinia necatrix* (44.26 Mb) and *Daldinia eschscholtzii* (35.81 Mb).

A linear regression analysis employing *Monosporascus* genomes as well as genomes from the Sordariales and other members of the Xylariales revealed a negative relationship between GC content and genome size (Figure 4). BUSCO, which is commonly used to assess genome completeness through the concept of highly conserved single-copy orthologs, was able to locate over 97% of the expected complete single-copy Ascomycota orthologs in each *Monosporascus* assembly (Table 2).

**Table 2:** Validation of gene annotation with BUSCO assessment with Ascomycota dataset (n = 1315)

Isolate	Complete (C)	Complete and single-copy (S)	Complete and duplicated (D)	Fragmented (F)	Missing (M)
*M. cannonballus* (CBS 609.92)	98.2%	98.0%	0.2%	0.8%	1.0%
*M. cannonballus* (CBS 586.93)	97.8%	97.4%	0.4%	1.1%	1.1%
*M. ibericus* (CBS 110550)	98.1%	97.9%	0.2%	0.8%	1.1%
*Monosporascus* sp. (5C6A)	98.1%	97.6%	0.5%	0.8%	1.1%
*Monosporascus* sp. (CRB-8-3)	98.4%	97.9%	0.5%	0.8%	0.8%
*Monosporascus* sp. (CRB-9-2)	98.3%	97.9%	0.4%	0.7%	1.0%
*Monosporascus* sp. (MG133)	98.4%	97.9%	0.5%	0.9%	0.7%
*Monosporascus* sp. (MG162)	98.4%	98.1%	0.3%	0.5%	1.1%
*Monosporascus* sp. (GIB2)	98.1%	97.6%	0.5%	0.8%	1.1%

### Bacterial associations

**Figure 5:**
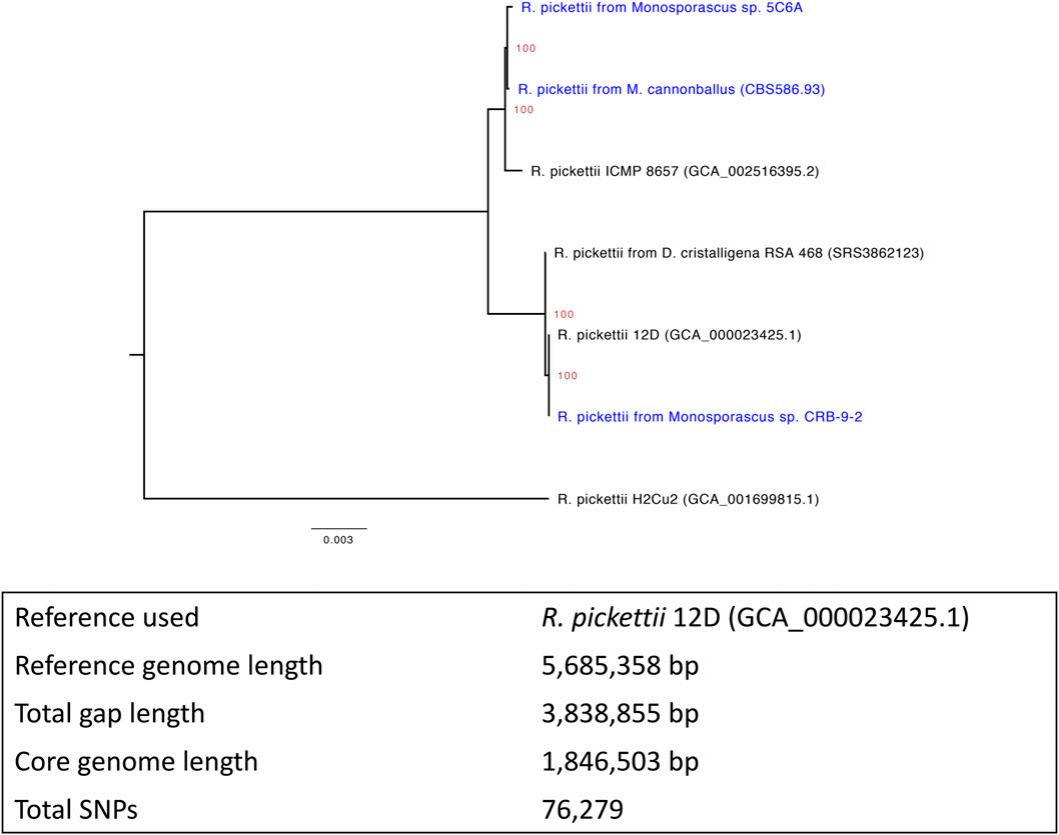
Maximum-likelihood tree displaying relationships between the *R. pickettii* lineages found in the *Monosporascus* (shown in blue) whole genome sequencing data and other closely related lineages. Bootstrap values (percent of 100 replicates) are displayed as red node labels. The relevant NCBI accessions for the non-*Monosporascus* lineages are listed in parentheses following the strain identifier. Genome assemblies were used for the 12D, ICMP 8657 and H2Cu2 isolates while SRA datasets were used for the *D. cristalligena* RSA 468 isolate. The PhaME summary statistics for this tree are presented in the table below.

During quality control filtering for NCBI submission we observed several contigs with strong hits to the bacterial species *Ralstonia pickettii*. Two of the *Monosporascus* assemblies contained nearly complete genomes for *R. pickettii*, and a third contained a large number of *R. pickettii* contigs. Three other assemblies contained a small (≤ 5) number of *Ralstonia* contigs, while the remaining four assemblies contained zero *Ralstonia* contigs. Phylogenetic analyses generated with PhaME (Shakya *et al.* 2020) using both raw Illumina reads and assembled contigs confirmed these bacterial sequences belong to distinct *R. pickettii* lineages, which is evidence against local contamination (Figure 5).

### Genome diversity

**Figure 6:**
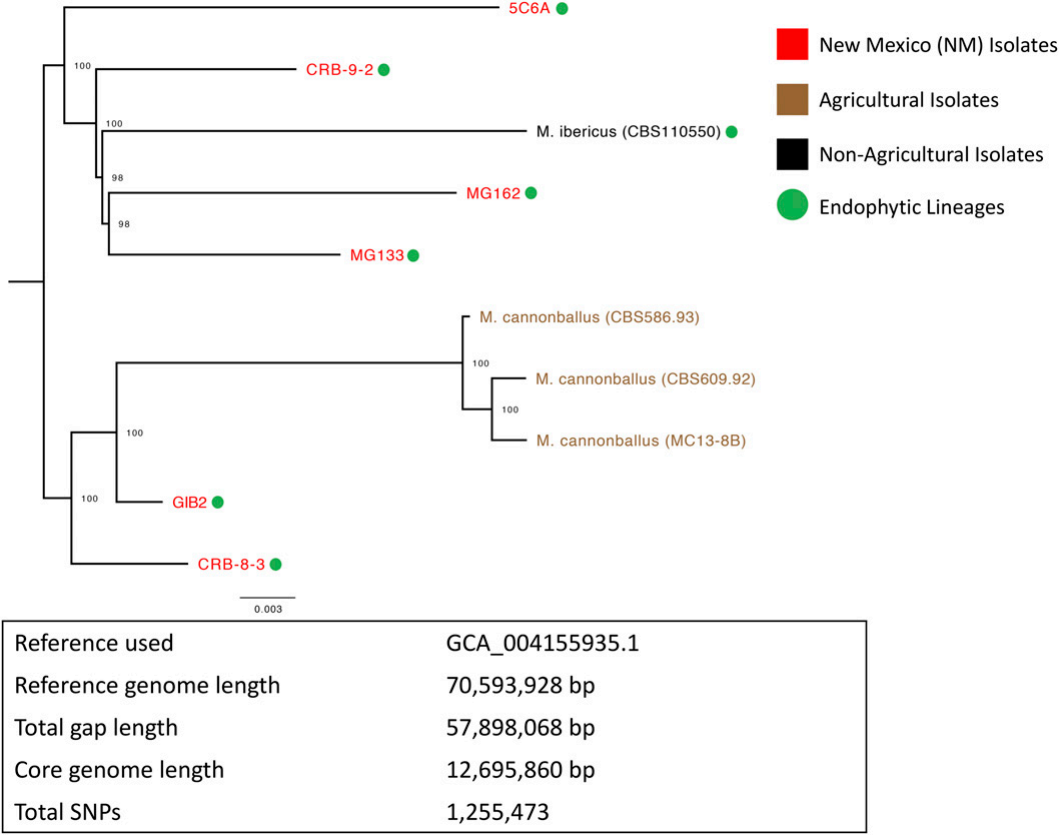
Maximum-likelihood tree generated in PhaME using reads obtained from whole genome sequencing of *Monosporascus* isolates and summary PhaME statistics. Bootstrap values (percent of 100 replicates) are shown as node labels. *Monosporascus* isolates from New Mexico are labeled in red, while agricultural isolates are labeled in brown. Endophytic isolates are labeled with green circles.

To better understand diversity within the genus *Monosporascus* we utilized the PhaME (Shakya *et al.* 2020) analysis tool to estimate phylogenetic relationships at the whole-genome, single-nucleotide polymorphism (SNP) level. PhaME was able to establish a core genome region of 12,695,860 base-pairs, and a total of 1,255,473 SNPs were used to determine the phylogenetic relationships between the *Monosporascus* isolates (Figure 6). This whole-genome phylogenetic analysis confirmed the high level of diversity initially observed with ribosomal RNA ITS analyses. There are close relatives of both *M. cannonballus* and *M. ibericus* among the New Mexican isolates. To gain a better understanding of the placement of *Monosporascus* within the Xylariales, a separate five-gene phylogenetic analysis was performed using the genes for beta-tubulin, MCM7, EF1α, RPB1 and RPB2 (Figure S1 & File S1), which are commonly used to examine phylogenetic relationships in fungi at various levels of divergence (James *et al.* 2006). The inclusion of several additional members of the Xylariales in this analysis suggests the *Monosporascus* isolates were most closely related to *Eutypa lata*, which is commonly associated with disease in grape vines and has been placed in the family Diatrypaceae (Acero *et al.* 2004).

Annotation of the *Monosporascus* genome assemblies indicate a predicted gene content similar to other members of the Sordariomycetes and Xylariales despite their inflated genome sizes (Figure 2B). There was no consistent relationship between genome size and predicted gene number among the *Monosporascus* assemblies or across the Xylariales in general (Table 3 & Figure 3B). Synteny comparisons among *Monosporascus* genomes and other members of the Xylariales identified regions of synteny as well as large regions lacking synteny. The genome from the GIB2 strain was used to examine in some detail the regions lacking synteny. This genome was used as a reference because it was the smallest *Monosporascus* genome and would therefore minimize noise from *Monosporascus*-specific variation (Table 3). We identified 401 contigs in the GIB2 assembly that did not show synteny with any of the non-*Monosporascus* assemblies listed in Table 3, although all of these contigs possessed synteny with at least one other *Monosporascus* genome. The combined size of these contigs was 12.04 Mb. Of these, 227 (10.82 Mb) contained at least one gene annotation with a total of 1,100 gene annotations across all 227 contigs, with a GC content of 35.11% (comparable to the average genome-wide GC content of 39.53% for all *Monosporascus* genomes). These 227 contigs appear to be enriched for genes involved in secondary metabolism. The remaining 174 contigs (1.22 Mb) had no predicted gene content and had a GC content of 24.76%, indicating the existence of AT-rich regions with no or obscured synteny with respect to members of the Xylariales outside the genus *Monosporascus*.

**Table 3:** Xylariales annotation, functional guild and assembly size comparison

Species (strain)	Predicted number of genes	Total length (Mb)	NCBI Accession	Functional Guild
*Monosporascus* sp. (GIB2)	11,783	70.59	QJNS00000000	Endophyte
*Monosporascus* sp. (MG133)	11,569	71.87	QJNT00000000	Endophyte
*Monosporascus* sp. (5C6A)	12,537	75.25	QJNW00000000	Endophyte
*Monosporascus* sp. (CRB-9-2)	12,001	76.10	QJNU00000000	Endophyte
*Monosporascus ibericus* (CBS110550)	11,063	87.25	*QJOB00000000*	Endophyte
*Monosporascus cannonballus* (MC13-8B)	10,641	89.29	*QJNZ00000000*	Pathogen
*Monosporascus* sp. (MG162)	11,881	89.46	*QJOA00000000*	Endophyte
*Monosporascus cannonballus* (CBS609.92)	10,682	89.62	QJNX00000000	Pathogen
*Monosporascus cannonballus* (CBS586.93)	11,585	91.50	*QJNY00000000*	Pathogen
*Monosporascus* sp. (CRB-8-3)	11,775	102.96	*QJNV00000000*	Endophyte
*Eutypa lata* (UCREL1)	11,685	54.01	AORF00000000.1	Pathogen
*Pestalotiopsis fici* (W106)	15,413	51.91	ARNU00000000.1	Endophyte
*Microdochium bolleyi* (J235TASD1)	13,338	38.84	LSSP00000000.1	Endophyte
*Rosellinia necatrix* (W97)	12,644	44.26	BBSO00000000.2	Pathogen
*Hypoxylon sp*. (CO27-5)	12,245	46.59	MDCL00000000.1	Endophyte
*Xylaria grammica* (IHI A82)	12,126	47.04	RYZI00000000.1	Endophyte

Substantial variation in predicted gene number existed among the three agricultural isolates from the single species *M. cannonballus*. *M. cannonballus* isolates from the southwestern United States, CBS609.92 and MC13-8B, were found to have very similar predicted gene numbers (10,682 and 10,641, respectively), but the strain from Egypt, CBS586.93, had a larger predicted genome size (91.50 Mb) and number of genes (11,585). Comparisons using the MMseqs2 search tool failed to find matches based on sequence identity for approximately 700 predicted amino-acid sequences from the Egyptian isolate when compared with either of the two agricultural isolates. Comparisons of protein similarity using CD-HIT-2D also indicated that the predicted amino-acid sequences in the southwestern *M. cannonballus* isolates were more similar among those isolates, based on identity, relative to the predicted amino-acid sequences found in the Egyptian isolate. The number of predicted protein sequences from the Egyptian isolate that did not pair with sequences from either of the southwestern isolates based on identity was larger than the differences between southwestern isolates.

### Sexual pheromone receptor homologs

Hidden Markov models (HMM) were used to construct predicted protein homologs for PRE-1 and PRE-2 (*Neurospora nomenclature*) using amino-acid sequences from diverse members of the Sordariomycetes, Pezizomycetes and Leotiomycetes. These HMMs successfully identified homologs of both PRE-1 and PRE-2 in all examined *Monosporascus* and Xylariales genomes (Figure S2 and S3). Homologs of the *pre-1* and *pre-2* from members of the Xylariales appear to be evolutionary distinct from other members of the Sordariomycetes.

### Carbohydrate-active enzymes and pathogenicity homologs

**Figure 7:**
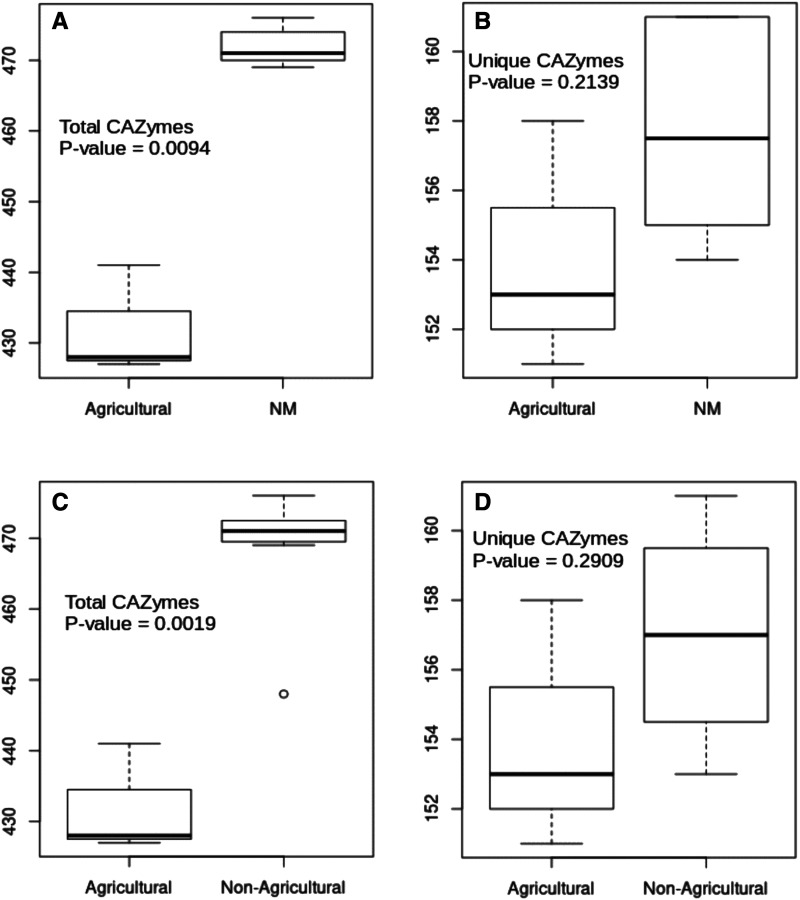
Boxplot comparisons of CAZyme content among the ten *Monosporascus* genomes examined. (A) and (B) Total and unique CAZyme content for the three agricultural *M. cannonballus* isolates and the six New Mexican *Monosporascus* isolates. (C) and (D) Total and unique CAZyme content for the three agricultural *M. cannonballus* isolates and the remaining seven non-agricultural *Monosporascus* isolates. The outlier point in (C) represents the *M. ibericus* isolate CBS110550.

Carbohydrate-active enzymes interact with a number of substrates that are common in plant cells, and it is predicted that some of these enzymes may facilitate plant-fungal interactions (Knapp *et al.* 2018). The New Mexican *Monosporascus* isolates have a significantly greater number of these predicted enzymes than do the agricultural *M. cannonballus* isolates (Figure 7A). Differences in the number of these predicted enzymes present between the New Mexico and agricultural *M. cannonballus* isolates were statistically insignificant when examining the presence of unique enzymes from each gene family (Figure 7B). When the non-agricultural *M. ibericus* isolate was included in these comparisons, the results were very similar (Figure 7C & 7D). Homologs of glycosidases comprised the majority of these predicted enzymes, and the genes for some enzyme types exhibited high copy numbers. The order Xylariales contains genera with species classified as pathogens as well as others classified as endophytes. Comparing profiles across these two ecological groups, there appears to be no observable relationship between either the total or unique number of genes for these predicted enzymes and primary ecological function (Table 4).

**Table 4:** Predicted carbohydrate-active enzymes from dbCAN2 analysis and number of pathogenicity and effector genes predicted by PHI-base analysis

Isolate (strain)	Predicted CAZymes*^a^*	Predicted Unique CAZymes*^a^*	Predicted Genes Associated with Pathogenicity	Predicted Effector Genes
*M. cannonballus**^b^* (CBS 609.92)	427	153	285	43
*M. cannonballus**^b^* (CBS 586.93)	441	158	301	59
*M. cannonballus**^b^* (MC13-8B)	428	151	285	45
*M. ibericus* (CBS 110550)	448	153	316	42
5C6A	474	154	320	56
CRB-8-3	469	161	322	51
CRB-9-2	471	158	323	58
MG133	476	161	331	41
MG162	470	157	330	47
GIB2	471	155	336	44
*Eutypa lata**^b^* (UCREL1)	494	158	305	40
*Daldinia eschscholzii* (EC12)	407	145	291	29
*Rosellinia necatrix**^b^* (W97)	444	166	317	47
*Microdochium bolleyi* (J235TASD1)	433	158	307	48

a Values were obtained from automated CAZyme annotations on protein sequences from each isolate. HMMER (E-Value < 1e-15, coverage > 0.35), DIAMOND (E-Value < 1e-102) and Hotpep (Frequency > 2.6, Hits > 6) were all utilized, and only candidates identified by at least two of these methods are represented.

b Pathogenic lineages.

**Figure 8:**
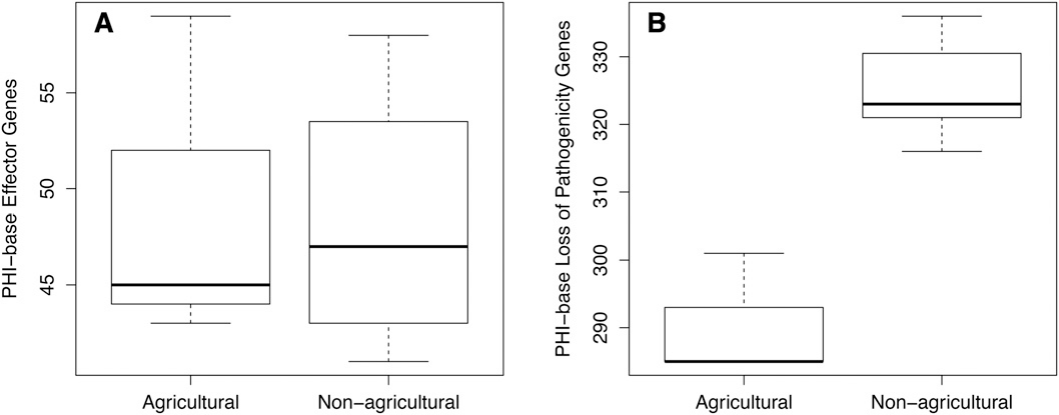
Boxplot comparisons of PHI-base gene content for the ten *Monosporascus* genomes examined. (A) Comparison of predicted effector gene content between the three agricultural *M. cannonballus* isolates and the other seven non-agricultural isolates (one *M. ibericus* isolate and six NM *Monosporascus* isolates). (B) Comparison of predicted content of genes associated with pathogenicity between the three agricultural *M. cannonballus* isolates and the other seven non-agricultural isolates. Genes represented in these figures had either an effector or loss of pathogenicity characterized top match in PHI-base with an e-value >= 1e-10.

The PHI-base database catalogs genes that have been experimentally verified to be involved in pathogenicity for both eukaryotic and prokaryotic pathogens that interact with plant hosts (Urban *et al.* 2017). The *Monosporascus* isolates from New Mexico contain a significantly higher number of pathogenicity homologs than do the *M. cannonballus* agricultural isolates (Figure 8B). This also raises a question about the function of these homologs and if perhaps they could have been altered to allow access to a broader host range or possibly encouraging a more mutualistic lifestyle.

Finally, we examined potential effector-protein genes within the genomes of *Monosporascus* isolates. Effector proteins are secreted by fungi to facilitate colonization of plant cells. The predicted effector protein content is very similar between the New Mexico and agricultural *M. cannonballus* isolates (Figure 8A). The inclusion of the non-agricultural *M. ibericus* isolate had a minimal effect on the comparison. Our comparison also included other members of the Xylariales and once again this analysis showed no obvious relationship between primary ecological function and the content of these two categories of PHI-base genes (Table 4).

## Discussion

The genus *Monosporascus* is diverse and contains several lineages. Complete genome sequences of ten *Monosporascus* isolates revealed larger than expected genome sizes and diverse genome content and organization. This diversity is apparent even among closely-related *M. cannonballus* isolates from different geographical origins. The sequencing design for this work was based on the assumption that these genomes would be similar in size to other members of the Xylariales, which would have resulted in an average coverage of around 100x. Given the much larger size of these *Monosporascus* genomes, the coverage is lower than what is typical for fungal *de-novo* genome sequencing projects. Despite their lower coverage, assemblies scored well in examinations commonly used to assess quality. Three different software packages designed to assemble microbial genomes produced *de novo* assemblies of similar size for each isolate. The consistency among these assembly methods and results supported the large genome estimates. Assemblies produced by SPAdes consistently had the largest N50 to number of scaffolds ratio, which was anticipated given previous benchmarking analyses that demonstrated proficiency with low coverage data (Bankevich *et al.* 2012).

While preparing our assemblies for submission we noticed that three of the *Monosporascus* assemblies contained a substantial number of bacterial contigs that were classified as *Ralstonia pickettii*. The genus *Ralstonia* belongs to the Burkholderiaceae, a family that contains known fungal endosymbionts (Uehling *et al.* 2017; Lastovetsky *et al.* 2018; Bonfante and Lanfranco 2019). Previous studies have demonstrated that *R. pickettii* can exist as a bacterial endosymbiont in fungi and that it is capable of promoting fungal pathogenesis through this intimate relationship (Itabangi *et al.* 2019). We initially considered the possibility that this bacterial signal was due to contamination, especially given that members of the Burkholderiaceae have been demonstrated to be present in extraction and sequencing reagents (Glassing *et al.* 2016). In nearly all demonstrated cases of reagent contamination, diverse taxa of bacteria are present, and even in instances involving amplification the relative percentages of sequences from these contaminating taxa are quite low in early serial dilutions (Salter *et al.* 2014). Given that our *Monosporascus* isolates were originally cultured on media containing antibiotics, the lack of other bacterial sequences at such abundances and the fact that *Ralstonia* sequences were not identified in all of the samples sequenced, it is unlikely that these sequences are the result of contamination during sample or sequencing preparation. While the *Monosporascus* isolates 5C6A and CRB-9-2 are closely related in our phylogenetic analysis (Figure 6), the *R. pickettii* signals detected in 5C6A and the more distantly related CBS 586.93 isolate appear to be more closely related. The *R. pickettii* signal detected in CRB-9-2 is closely related to the environmental lineage *R. pickettii* 12D and the *R. pickettii* signal detected in the whole genome sequencing project of *D. cristalligena* RSA 468. The size of the core genome lengths detected by PhaME (∼33%) suggest the examined *R. pickettii* genomes are quite evolutionarily diverse.

Our previous work demonstrated that the canonical mating type regions are either missing or highly divergent in these *Monosporascus* genomes and all other members of the Xylariales examined (Robinson and Natvig 2019). Homologs of the *Neurospora crassa* sex pheromone receptor genes *pre-1* and *pre-2* are involved with mating behavior in species of *Neurospora* and diverse fungi, and their expression is typically under the control of mating-type genes (Kim and Borkovich 2004; Jones and Bennett 2011; Kim *et al.* 2012), making them logical targets for subsequent analysis of downstream effects caused by genetic changes in the mating-type region. The presence of these pheromone receptor genes indicates that downstream signaling pathways related to sexual reproduction may be conserved in the Xylariales, despite the absence of canonical mating-type regions. We acknowledge, however, that fungal pheromone receptors have been reported with atypical expression patterns and altered functions in the homothallic species *Gibberella zeae* (Lee *et al.* 2008), and a homolog of the PRE-2 receptor in *Fusarium oxysporum* has been reported to be involved in host-pathogen recognition (Turrà *et al.* 2015). Therefore, the presence of these receptor genes in species of *Monosporascus* cannot be taken as proof of their involvement in mate recognition.

Endophytic isolates of *Monosporascus* from New Mexico associate with a broad range of hosts, and evidence from molecular and culture studies suggest that this broad host range can apply to individual terminal clades within the genus. This raises questions regarding whether presumed endophytic lineages differ from pathogenic lineages with respect to specific genes or groups of genes. We therefore compared the genomes of endophytic and pathogenic isolates within the genus *Monosporascus* and also across genera within the Xylariales with respect to genes for carbohydrate-active enzymes, genes known to be involved in pathogenicity in certain fungi, and genes for effector proteins that facilitate colonization of plant tissues. It remains unclear if the *Monosporascus* isolates from New Mexico are capable of causing disease, but they contain more predicted genes associated with pathogenesis and host plant interactions than their agricultural relatives. In any event, *Monosporascus* isolates are capable of associating with a broad diversity of plant hosts, suggesting they will be important in understanding mechanisms of plant-fungal interactions.
